# The Interaction between Joint Inflammation and Cartilage Repair

**DOI:** 10.1007/s13770-019-00204-z

**Published:** 2019-07-26

**Authors:** Peter M. van der Kraan

**Affiliations:** Experimental Rheumatology, Department of Rheumatology, Radboudumc, Radboud Institute for Molecular Life Sciences (RIMLS), Geert Grooteplein 26, 6525 GA Nijmegen, The Netherlands

**Keywords:** Cartilage repair, Fracture repair, Chondrogenesis, Inflammation, Innate immunity

## Abstract

**Background:**

Articular cartilage lesions occur frequently but unfortunately damaged cartilage has a very limited intrinsic repair capacity. Therefore, there is a high need to develop technology that makes cartilage repair possible. Since joint damage will lead to (sterile) inflammation, development of this technology has to take into account the effects of inflammation on cartilage repair.

**Methods:**

A literature search has been performed including combinations of the following keywords; cartilage repair, fracture repair, chondrogenesis, (sterile) inflammation, inflammatory factors, macrophage, innate immunity, and a number of individual cytokines. Papers were selected that described how inflammation or inflammatory factors affect chondrogenesis and tissue repair. A narrative review is written based on these papers focusing on the role of inflammation in cartilage repair and what we can learn from findings in other organs, especially fracture repair.

**Results:**

The relationship between inflammation and tissue repair is not straightforward. Acute, local inflammation stimulates fracture repair but appears to be deleterious for chondrogenesis and cartilage repair. Systemic inflammation has a negative effect on all sorts of tissue repair.

**Conclusion:**

Findings on the role of inflammation in fracture repair and cartilage repair are not in line. The currently widely used models of chondrogenesis, using high differentiation factor concentrations and corticosteroid levels, are not optimal. To make it possible to draw more valid conclusions about the role of inflammation and inflammatory factors on cartilage repair, model systems must be developed that better mimic the real conditions in a joint with damaged cartilage.

## Introduction

Repair of an injured organ occurs when the damaged tissue is removed, lesions are healed, and inflammation is resolved. Regeneration, on the other hand, is defined as the reconstitution of the damaged tissue in a scar-free manner regaining all the initial functions of the tissue or organ. The distinction between what drives either repair or regeneration is nowadays an important subject of research to elucidate what signaling networks drive the healing process to either regeneration or (imperfect) repair. An important research question is if we can adjust these signaling networks for our own good to stimulate more efficient repair or even regeneration of tissues and organs in humans? A central element of the healing process is the concomitant inflammatory response that is an inevitable consequence of all tissue damage.

Inflammation has in general a bad reputation and is regarded as a process to avoid or inhibit. However, in the context of evolution it is very improbable that nature “invented” such a sophisticated and well-developed system to damage the individual in which it is occurring. Inflammation is both an important host defense mechanism and as well as a trigger to repair damaged tissues. However, there is a general perception that if inflammation does not resolve, neither efficient repair nor regeneration will occur leading to sustained functional problems.

In this review the interplay between repair and inflammation is discussed in the context of tissue damage without infection (sterile inflammation) focusing on the repair of cartilage defects. Most studies on the relationship between tissue healing and inflammation investigate the healing of soft tissue wounds. However, soft tissue wounds or cartilage lesions are very different in their cellular composition, matrix components and repair response. Although dissimilar from cartilage healing, bone fracture healing can be expected to have overlapping attributes with cartilage repair, at least partly with healing of full thickness defects, since chondrogenesis and cartilage formation is a crucial aspect of both fracture repair and repair of cartilage full thickness defects. Therefore, in this narrative review, the relationship between inflammation and repair will be discussed in short in a general setting and more specific in fracture repair. Furthermore, this review discusses what is known about the role of inflammation and inflammatory factors in repair of cartilage lesions, including effects of inflammation on chondrogenesis.

## Damage and inflammation

Inflammation and repair are not separate entities but a continuum with common actors that have differential roles at different stages of the biological response initiated by tissue damage. Sterile inflammation, as a result of acute or chronic tissue damage, results in the release of damage-associated molecular patterns (DAMPS) that initiate the inflammatory response to injury. DAMPS are endogenous molecules that indicate tissue damage and are actively or passively released from injured cells and extracellular matrix [1]. These DAMPS activate resident cells triggering these cells to release chemotactic factors that attract inflammatory cells to the wound site [2, 3]. Activation of Toll-Like Receptors (TLRs) on cells leads to chemokine release and attraction of inflammatory cells. Since DAMPS also induce chemokine release by the cells that enter the wound site this results in a positive feedback loop that only ends when the amount of activating DAMPS is ebbed [3]. The cells invading the wound site do not only play an important role in the initiation of the inflammatory response but also in the resolution of inflammation.

The first inflammatory cells that are attracted to the site of damage are neutrophils. Neutrophils are attracted to the wound site by a chemotactic gradient of molecules released by damaged tissues and activated cells and platelets. In zebra fish, reactive oxygen species (ROS) and Interleukin-8 have been shown to be involved in the attraction of neutrophils to the wound site [4]. Inhibition of ROS production impaired the initial recruitment of neutrophils to damaged tissue. Resolution of inflammation is thought to involve neutrophil apoptosis. However, in zebra fish it has been shown that less than 3% of the neutrophils undergo apoptosis at the wound site [5]. An alternative mechanism to eliminate neutrophils from the wound site appears to be at play, reverse migration of neutrophils from damaged tissue back toward the vasculature [6].

Neutrophils are also recruited to damage sites by the proinflammatory cytokine Interleukin-17A (IL-17A). Interestingly, IL17A is also produced by neutrophils [7]. IL-17A-knockout mice show enhanced skin wound healing and decreased neutrophil accumulation compared to wild-type mice [8]. In line with this, administration of recombinant IL-17A led to delayed healing and enhanced neutrophilic accumulation. Furthermore, the treatment of IL-17A-administered mice with a neutrophil elastase inhibitor reestablished repair to the same level as that of WT mice. These results indicate that IL-17A impairs wound healing and suggest that neutrophilic inflammation caused by IL-17A may be associated with impaired wound healing in skin.

Macrophages represent the major population of resident phagocytes in tissues under basal conditions. During health, tissue-resident macrophages can be found in different organs and have vital roles in tissue development and maintenance of homeostasis. Upon tissue damage and inflammation, macrophages can arise from circulating monocytes that infiltrate the wound site. Local tissue damage leads to the swift recruitment of blood-derived, and neighboring-tissue derived, monocytes that develop into macrophages. These Infiltrating monocytes/macrophages are the first cells that after the neutrophils penetrate the damaged tissue. These infiltrating cells contribute to the total pool of macrophages found at the wound site after injury, together with the original tissue-resident pool. Macrophages sense tissue damage and remove debris and apoptotic cells from the wound site but also contribute to initiation and progression of tissue repair. In several animal models, amongst others in mice, guinea pig and zebra fish models, it has been shown that depletion of macrophages impairs regeneration [9–11].

Monocytes represent a highly plastic and dynamic cell type that contributes to the local pool of macrophages. Whether monocyte-derived macrophages and tissue-resident macrophages have similar functions under inflammatory conditions is not elucidated yet.

Macrophages undergo phenotypic changes in response to injury and repair. Macrophages are historically classified according to their *in vitro* mode of activation as classically activated macrophages (M1) or alternatively activated (M2) macrophages [12]. M1 macrophages show pro-inflammatory properties by producing pro-inflammatory cytokines such as tumor necrosis factor alpha (TNF-alpha) and interleukin 1β (IL-1β). M2 macrophages, in contrast, display anti-inflammatory and repair features by expressing interleukin 10 (IL-10) and transforming growth factor β (TGF-β). Recently, the concept has been developed that this classification is too simplistic and it has undergone substantial revision. A, new complex classification model has been proposed based on activating stimuli, ontogeny, activation markers and experimental setting owing to the far more complex and distinct phenotypes of macrophages in various tissues [13].

Also, other cells than neutrophils and monocytes migrate to the damage site as have been found in murine models of sterile inflammation. Dendritic cells and lymphocytes play their part in sterile inflammation and repair. Dendritic cells are activated as a response to cell death and promote T-lymphocyte responses to antigens presented by these cells [14]. It has been shown that under sterile inflammatory conditions memory T cells (CD8) are generated [15]. Not only T cells but also B cells are involved in sterile inflammation. In the spleen a B cell response is initiated through sterile inflammation [16]. Furthermore, it has been shown that after myocardial infarction T and B cells infiltrate the wound area [17]. Murine peritonitis induced by injection of dead yeast particles results in migration of innate lymphoid cells at the site of inflammation [18]. What is more, invariant natural killer T cells populated sterile hepatic wound in the late stage after damage releasing the anti-inflammatory cytokine interleukin-4 (IL-4) that was shown to improve wound healing [19]. Interleukin-4 producing macrophages activating invariant natural killer T cells to produce cytokines including IL-4, IL-13, and interferon-gamma have been detected in this system [20]. The production of IL-4 and IL-13 appears to be important to resolve inflammation since deficiency of IL-4, the IL-4 receptor or natural killer cells increased peritonitis severity. These results indicate that efferocytosis-induced IL-4 production and activation of IL-4-producing invariant natural killer T cells by macrophages appear to be required to resolve sterile inflammation and promote tissue repair.

Starting with the infiltration of neutrophils and ending with invariant natural killer T cells it is clear that repair and inflammation cannot be considered as separate entities but that these are all aspects of a continues process. The cells and cytokines involved have not a single biological role but have differential roles depending on the context and the stage of damage and repair. A downside of this realization is that a simple solution to boost repair by just inhibiting inflammation is not a likely option.

## Fracture repair and inflammation

Depending on the type of cartilage lesions, chondral or full thickness defects, the repair reaction is completely different from soft tissue repair or has a number of overlapping attributes. This raises the question what we can learn from soft tissue repair that helps us in improving cartilage repair. A lesion that is more closely related to cartilage repair than soft tissue repair is fracture healing. How inflammation affects fracture repair can give us clues about the relationship between inflammation and cartilage repair.

Immediately after a bone is fractured a haematoma is formed. This haematoma consists of cells from the bone marrow, periosteum and the blood. The fracture triggers an inflammatory reaction which is essential to start the healing to progress. The inflammatory reaction causes the haematoma to coagulate between the fracture ends forming a template for callus formation. The action of proinflammatory molecules is critical to initiate the response of bone. It has been shown that absence of TNF-alpha signaling in TNF-receptor knockout mice leads to a delay in both intramembranous and endochondral bone formation [21]. This observation indicates that inflammatory TNF-a signaling makes the repair process possible, maybe by stimulating mesenchymal stem cell recruitment and differentiation [21].

In humans it is known that the sterile inflammation after certain surgical procedures, such as hip replacement, induces ectopic bone formation which can be prevented by treatment of patients with NSAIDs [22]. In line with this observation is the finding that in mice COX-2 is important in bone healing [23]. COX-2 null mice have been shown to have a highly reduced capacity for bone repair. Furthermore, in transplantation studies in mice using cortical bone grafts it was shown that COX-2, produced by infiltrating inflammatory cells and periosteal progenitors, is essential in the early process of bone repair [24].

Fra-1 is a component of the transcription factor activator protein-1 (AP-1) and a factor known to be involved in the regulation of inflammation [25]. In Fra1 transgenic mice chondrogenesis around the fracture site was impaired, resulting in accumulation of fibrous tissue, which interfered with efficient bone healing [26]. Unexpectedly, immediately after fracture, induction of the inflammatory mediators TNF-alpha, Interleukin (IL)-6, and COX-2 was significantly suppressed in Fra-1 transgenic mice indicating reduced inflammation leads to impaired bone healing. Administration of prostaglandinE2 (PGE2) to the fracture site using a slow-release carrier significantly reduced accumulation of fibrous tissue in Fra-1 transgenic mice and chondrogenesis was partially restored. These data suggest that the Fra-1-containing transcription factor AP-1 inhibits fracture healing through suppression of inflammation-induced chondrogenesis.

The above described findings indicate that early and local inflammation stimulates bone healing. This appears to be totally different for systemic inflammation. For instance, systemic inflammation induced by systemic lipopolysaccharide (LPS) administration in a mouse model for femoral fracture repair impaired bone healing [27]. This effect was associated with decreased revascularization and bone turnover, and by increased abundance of macrophages. Systemic treatment of mice with a soluble glycoprotein 130 fusion protein (blocking IL-6 trans signaling) or an anti-IL6 antibody (blocking global IL-6 signaling) showed that selective inhibition of IL-6 trans-signaling improved the fracture healing while global IL-6 inhibition did not affect fracture healing. These data suggest that classic signaling might have beneficial effects on bone repair after injury [28]. Administration of the NSAID diclofenac in the inflammatory phase of bone repair in mice with or without lipopolysaccharide-induced systemic inflammation showed that administration of NSAIDs in the early stage of fracture repair inhibits bone healing, with reduced osteoblast, osteoclast, and macrophage activity, and even exacerbated the negative effects of systemic inflammation on the healing process [29]. It seems clear that systemic inflammation, in contrast to local inflammation, impairs bone healing but the underlying mechanisms remain poorly defined.

## Inflammation and cartilage repair

Cartilage damage can have different features. Chondral cartilage defects are confined to the superficial cartilage without damaging the subchondral bone at the moment of trauma. Since cartilage is not innervated and avascular, a chondral defect does not lead to a genuine repair response as can be seen after soft tissue injury. These chondral defects result in distorted joint mechanics, altered distribution of cartilage loading and stresses, and degenerative changes in the long run. In many trauma patients chondral defects are not the sole lesion in the joint, cruciate ligament rupture or meniscal tears can go alongside. As a result of tissue damage inflammatory pathways will be activated in the traumatized joint that will contribute to or interfere with joint homeostasis and repair.

A full thickness defects is characterized by a cartilage lesion in combination with a lesion into the subchondral bone. Since this leads to the formation of a blood cloud, repair is initiated, and the defect will be ultimately filled with biomechanically inferior fibrocartilage. Normal hyaline, articular cartilage is not formed, but in many cases the fibrocartilage can prevent disability in the short run but frequently lead to disability later.

The most widespread damaged cartilage can be found in the joint of patients with osteoarthritis (or other types of arthritis). Osteoarthritis is more generalized cartilage damage compared to the focal defects in chondral and full thickness lesions although also osteoarthritis can be focal in the early stage. Furthermore, osteoarthritis is considered a disease of the whole joint since changes are not confined to the articular cartilage but also other joint tissues, e.g. subchondral bone and synovium, are affected. Since cartilage damage in an OA joint can be widespread repairing cartilage damage in an OA joint is even a greater challenge than cartilage repair in a focal defect.

Articular cartilage has a very limited intrinsic repair capacity leading to progressive joint damage when injured. Mesenchymal stromal cells (MSC) are a promising tool for tissue engineering of articular cartilage. The *in vitro* system of chondrogenic differentiation of mesenchymal stem cells in a three-dimensional culture system combined with the addition of differentiation factors has widely been used as model for chondrogenesis and cartilage formation. In general, this system does not lead to the formation of stable articular cartilage-like tissue. The most efficient factors to induce chondrogenesis in this system include members of the transforming growth factor beta superfamily in combination with dexamethasone [30]. Combinations of these factors induce the expression of chondrocyte specific genes in MSC, but these factors are also highly anti-inflammatory. Both TGF-beta and corticosteroids belong to the most potent anti-inflammatory molecules known [31–33]. Using these molecules in a culture system of chondrogenesis it is not unexpected that the effect of inflammatory factors will be underestimated in this culture model. The effect of additional inflammatory or anti-inflammatory factors being easily overruled by dexamethasone. We have observed that the effect of anti-inflammatory compounds on blocking the inhibitory effect of inflammatory cytokines or inflamed conditioned medium can be undetectable in the presence of dexamethasone, but clearly present without (data not shown). One has to be aware therefore that both the effect of inflammatory or anti-inflammatory compounds can be underestimated in the currently most used chondrogenic culture systems.

The repair of cartilage defects being either chondral, full-thickness or as a results of (osteo)arthritis has to be achieved in a milieu with tissue damage and as a consequence of this with minor or severe inflammation [34, 35]. The amount of inflammation will differ considerably between patients, but at least slight inflammation will be present in all patients. Cartilage damage will release DAMPS that will activate synovial lining cells via TLRs or other DAMP receptors [36]. After an acute joint trauma cytokine levels increase, and these will be elevated if damage is not fully repaired [37, 38]. In the case of cartilage damage, it is known that spontaneous full repair in human adults is rare or even considered to be absent. To repair cartilage, strategies have been developed using either chondrocytes or progenitor cells but the presence variable inflammation in the damaged joint have to be taken into account to make long term repair successful.

Children with chronic inflammatory disease exhibit disturbed growth [39, 40]. Longitudinal growth depends on normal growth plate functioning with strictly regulated chondrocyte proliferation and differentiation, processes that are also crucial in successful cartilage repair. Proinflammatory cytokines affect normal growth plate function amongst others by affecting Insulin-like growth factor I (IGF-I) signaling. Children with chronic inflammation have elevated levels of IL-6 and TNF-alpha in their blood. These children regularly show reduced systemic levels of IGF-I. Furthermore, mice that over express TNF-alpha show growth retardation. Inflammatory cytokines, such as TNF-alpha and IL-1, inhibit differentiation of growth plates chondrocytes and longitudinal growth [41]. Proinflammatory cytokines IL-1, IL-6 and TNF-alpha inhibited chondrogenesis in an *in vitro* growth plate model using the mesenchymal chondrogenic cell line RCJ3.1C5.18 [42]. These cytokines inhibited IGF-I-enhanced chondrocyte differentiation by blocking IGF-I-specific signaling pathways.

In particular the duration of exposure to proinflammatory cytokines appears to be important. A short exposure has little effects while long exposure leads to a severe and irreversible reduction in growth [43]. These studies indicate that proinflammatory cytokines interfere with normal chondrocyte differentiation. Although differentiation of growth plate chondrocytes and cartilage repair is different there is an overlap in biological processes and one could assume that the negative effect of proinflammatory factors on growth plate chondrocytes has its equivalent in repair of articular cartilage using progenitor cells.

In damaged joint inflammatory factors are produced locally by cells in the synovial fluid or released in the synovial fluid from surrounding tissues. Rodrigo et al. [44] studied the effect of synovial fluid from knees with a traumatic defect on chick limb chondrogenesis. Synovial fluid from most of these patients stimulated chondrogenesis (65%), the remainder had an no inhibitory effect (24%) or was without effect (11%). Synovial fluid from chronically injured knees was always inhibitory. In a study of Yang using redifferentiaton of human chondrocytes as a model, synovial fluid from injured knee joints inhibited cartilage-related matrix synthesis [45]. Chondrogenesis of subchondral mesenchymal cortico-spongious progenitor cells was inhibited by synovial fluid from rheumatoid arthritis patients but not by synovial fluid from osteoarthritis patients or controls [46]. Apparently, synovial fluid contains chondrogenesis promoting factors, but the positive action of these factors can be overruled by inflammatory factors present.

The source of these factors is currently not fully elucidated. This can be the inflamed, activated synovium but also other joint tissues can contribute. The infrapatellar fat pad (IPFP) has been described as a source for inflammatory factors [47]. Conditioned medium from the intrapatellar fat pad from injured knee joints inhibits cartilage formation in human MSC [48]. No differences were observed between traumatically-injured or osteoarthritic joints. Furthermore, conditioned medium from macrophages derived from the infrapatellar fat pad also decreased chondrogenesis. Conditioned medium obtained from cultures of M1 macrophages decreased chondrogenesis of human MSC while this was not the case by medium from M2 macrophages [49]. These results indicate that M1 macrophages are the cells in the joint that release factors that inhibit chondrogenesis.

In the presence of IL-1, the chondrogenic differentiation and cartilage formation of bone marrow-derived MSC is inhibited which can be partly overcome by culturing under hypoxic conditions [50, 51]. In a study by Wehling et al. [52], it was shown that both IL-1 and TNF-alpha inhibited chondrogenesis in MSC in a dose dependent way which correlated with activation of NF-kappaB. Specific inhibition of NF-kappaB blocked the activation of NF-kappaB and rescued chondrogenesis. Similar findings were reported by Han et al. IL-1beta and TNF-alpha inhibited the expression of chondrogenic-related genes in osteoarthritic synovium-derived stem cells, which was counteracted by knockdown of NF-kappaB and C/EBPbeta [53]. In our studies we showed that IL-1 alpha was more potent than TNF-alpha in inhibiting chondrogenesis of bone marrow-derived human MSC. In addition, incubation of MSC with conditioned medium of osteoarthritic synovium strongly inhibited cartilage formation and this could be partially overcome by blocking IL-1 but not by blocking TNF-alpha [51]. Furthermore, the negative effects of OA synovium conditioned medium could to a certain extent be overcome by blocking JAK signaling using tofacitinib or TAK-1 signaling using oxozeaenol [54]. MSCs cannot only be derived from bone marrow but also from synovial fluid. Interferon-gamma and TNF-alpha decreased cartilage formation in equine MSC from either bone marrow or synovial fluid [55].

In addition, also in human adipose derived mesenchymal stem cells chondrogenesis is inhibited by IL-1 [56]. Addition of diallyl disulfide blocked the reduction of chondrocyte marker gene expression by IL-1 in this system. Furthermore, expression of anti-oxidant enzymes was enhanced while ROS production and NF-KappaB signaling was reduced. Also, melatonin has been shown to reduce ROS accumulation and to inhibit the negative effects of IL-1 and TNF alpha on chondrogenesis in MSC [57]. Overall it clear that chondrogenesis of progenitor cells of various sources is inhibited by inflammatory factors pointing towards a negative effect of inflammation on chondrogenesis and new cartilage formation.

Inhibition of chondrogenesis is not only studied using human progenitor cells but also cell lines have been used and MSC from other species. Incubation of ATDC5 cells, a clonal murine chondrogenic cell line, in a chondrogenic culture system with IL-1 results in inhibition of chondrogenesis [58]. Moreover, the protein 14-3-3η is induced by TNF-alpha and found in the serum and synovial fluid of patients with joint inflammation [59]. Chondrogenesis of ATDC5 cells was inhibited by TNF but also by overexpression of 14-3-3η, while silencing of 14-3-3η stimulated chondrogenesis [59]. As similar a effect was observed by IL-6, a factor highly elevated under inflammatory conditions. The expression of chondrogenic differentiation marker genes was reduced by IL-6 in ATDC5 cells and this was blocked by an anti-IL6 receptor antibody [60]. In a study using murine bone marrow-derived MSC a similar effect of IL-6 was observed [61]. Also, these studies indicate that inflammatory factors block chondrogenesis and will be inhibitory in the context of cartilage repair.

## Concluding remarks

The relationship between inflammation and tissue repair is not simple and uncomplicated. Soft tissue repair is quite different from cartilage repair and therefore the role of inflammation in soft tissue repair is hard to extrapolate to cartilage repair. Surprisingly, also in fracture repair, which is thought to have a number of characteristics similar to cartilage repair, the role of inflammation seems to differ from cartilage repair. In fracture healing it appears that local inflammation is necessary for efficient repair and blocking inflammation in the initial stage of repair is deleterious. In contrast, in most (*in vitro*) models of chondrogenesis and cartilage formation by progenitor cells blocking inflammation enhances chondrogenesis and cartilage formation. Furthermore, systemic inflammation appears to have a negative effect on all varieties of tissue repair.

The discrepancy between fracture repair and cartilage repair is unexpected since chondrogenesis is an important aspect of both processes. The difference can be due to several reasons. Studies investigating the role of inflammation in fracture repair are mostly *in vivo* studies involving a whole organism while studies investigating the role of inflammation in cartilage are mostly *in vitro* studies. It will be clear that the *in vivo* test system is more complex involving feature that are missing in most *in vitro* studies, such as loading and a complex interaction between different cell types. Furthermore, as pointed out earlier, the currently used models of chondrogenesis, high growth factor concentrations and high concentrations of corticosteroids, are quite nonphysiologic compared to the conditions in a joint with a cartilage lesion. To really be able to draw valid conclusions about the role of inflammation and inflammatory factors on cartilage repair a *in vitro* model system should be developed that more closely resembles the actual conditions in a damaged joint.
